# Standing the test of time: total aortic remodeling 13 years after TEVAR for acute type B aortic dissection

**DOI:** 10.1007/s12055-023-01586-5

**Published:** 2023-09-04

**Authors:** Matthias Niklas Hagedorn, Katrin Meisenbacher, Denis Skrypnik, Moritz Sebastian Bischoff, Dittmar Böckler

**Affiliations:** Department of Vascular and Endovascular Surgery, Heidelberg University Hospital, Im Neuenheimer Feld 420, 69120 Heidelberg, Germany

**Keywords:** Thoracic endovascular aortic repair, Type B dissection, Long-term follow-up, Descending aorta, Endovascular treatment/therapy

## Abstract

Long-term outcome after thoracic endovascular aortic repair (TEVAR) of acute type B aortic dissection (aTBAD) is still underreported in current literature. This case report shows persistence of aortic remodeling without secondary complication or need of reintervention 13 years after TEVAR. A 45-year-old woman was referred to the emergency room with aTBAD. Due to early diameter progression in combination with therapy-refractory pain and uncontrolled hypertension, TEVAR was performed. Hereafter, the patient showed complete remodeling of the descending thoracic aorta without persistent false lumen perfusion in this segment and with stable true and false lumen diameter in the untreated abdominal segment for a 13-year period. No aortic-related reintervention was needed. With contemporary devices and adapted therapy, TEVAR seems able to treat complex thoracic disease. Long-term follow-up (FU) is mandatory to monitor the efficacy and durability of endovascular treatment in aortic disease.

## Introduction

Endovascular treatment strategy in acute type B aortic dissection (aTBAD) remains a topic of debate with respect to long-term outcomes, which is only rarely available in current literature.

Reports on short-term success do exist, regardless the technique being used. More important here is the question of (1) durability of the prosthesis and (2) maintenance of the therapeutic morphological success.

Herein, we describe the history of a 45-year-old woman with aTBAD, treated with thoracic endovascular aortic repair (TEVAR) 13 years ago. The patient was adherent to the follow-up protocol since that date, showing no secondary expansion of the dissected abdominal aortic segment.

## Case report

**Fig. 1:**
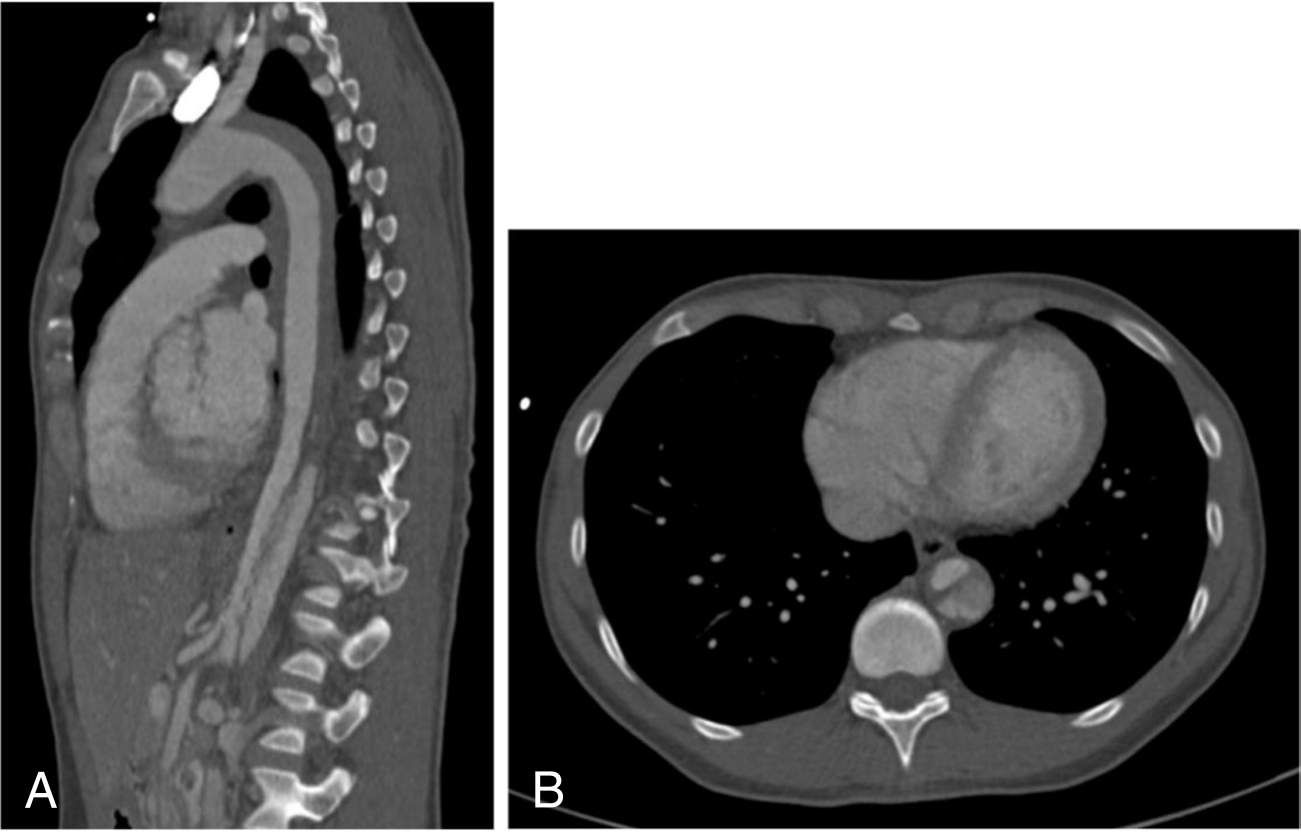
Initial CTA (**A** sagittal, **B** axial) showing an intramural hematoma followed by a true aortic dissection reaching from the left subclavian artery to the aortic bifurcation. CTA, computed tomography angiography

A 45-year-old woman was referred to the authors’ emergency room in October 2009 with suspected aortic dissection after external rule out of myocardial infarction. The patient initially suffered from acute thoracic pain, hypotension, and a self-limiting period of paraparesis lasting about 15 min. At the time of presentation, the patient was asymptomatic. The initial computed tomography angiography (CTA) scan performed in the emergency room showed an intramural hematoma (IMH), reaching from the left subclavian artery (LSA) throughout the entire thoracic aorta, without any signs of classical entries, but several small intramural blood poolings. In the visceral segment, however, there was a dissection, with the first entry at the level of the celiac trunk, reaching down to the aortic bifurcation (Fig. 1).

Imaging, laboratory results, and clinical examination were unsuspicious for any malperfusion.

Following guideline recommendations, the patient was admitted to the intensive care unit for conservative treatment [1].

Two days later, a CTA was performed due to recurrent thoracic pain and therapy-resistant hypertension, which revealed an IMH progression to a full dissection with false lumen (FL) expansion by 8 mm and a new left-sided pleural effusion. Multilinear reconstruction and centerline evaluation (OsiriX PRO, acyan, Rochester, USA) showed a “gothic” arch and a 12-mm-diameter mismatch between the proximal (24 mm) and distal landing zone (12 mm).

After planning and sizing, it was decided to perform TEVAR in order to prevent further progression. Due to its low radial force and favorable conformability to the aortic arch, a GORE® TAG conformable thoracic stent graft (CTAG) was chosen, which had just received Conformité Européenne (CE) approval for TEVAR in Europe platform.

The procedure was performed under general anesthesia. Before TEVAR, LSA transposition was performed to create a sufficient landing zone and to minimize the risk of spinal cord ischemia [1].

Subsequently, the right common femoral artery was exposed. An 8-French sheath was inserted via puncture. After a Terumo wire was established into the infrarenal aorta, a pigtail catheter was used to perform a staged angiography. After visualization of the aortic arch and marking the orifice of the left common carotid artery (LCCA), a stiff wire was inserted. After changing onto a 20-French Gore DrySeal sheath (Gore Medical, USA), the first CTAG device (26 mm × 26 mm × 100 mm, 10% oversizing) was positioned and released distal to the LCCA orifice under rapid pacing and distally extended by three other CTAG prostheses (26 mm × 21 mm × 100 mm and two times 21 mm × 21 mm × 100 mm, 10% oversizing) ending right before the orifice of the celiac trunk.

Following the institutional protocol, prophylactic cerebrospinal fluid drainage was established pre-procedural, due to long segment coverage and the patient history of paraparesis, in terms of spinal cord protection.

After a short period of postoperative surveillance, the patient was transferred to the normal ward with no signs of neurological deficit and under normotensive blood pressure control. Before discharge, a postoperative CTA scan was performed, confirming correct position of the device as well as FL thrombosis in the stent-covered aortic segment. There was no sign of early expansion. The patient was discharged after a total of 20 days without any disabling restrictions.

Since that time, the patient was adherent to the local follow-up protocol (FU protocol). Repetitive CTA scans showed a complete remodeling of the descending thoracic aorta with constant aortic diameters despite persistent FL perfusion in the abdominal segment without any early diameter progression.

**Fig. 2:**
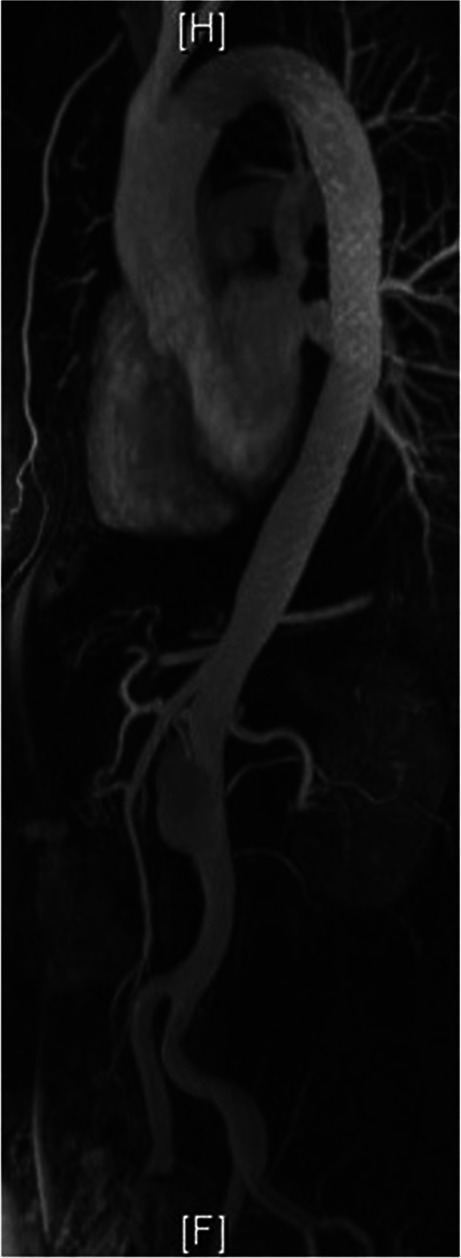
3D reconstruction of an MRA 7 years after TEVAR implantation with almost complete aortic remodeling. 3D, three-dimensional; MRA, magnetic resonance angiography; TEVAR, thoracic endovascular aortic repair

**Fig. 3:**
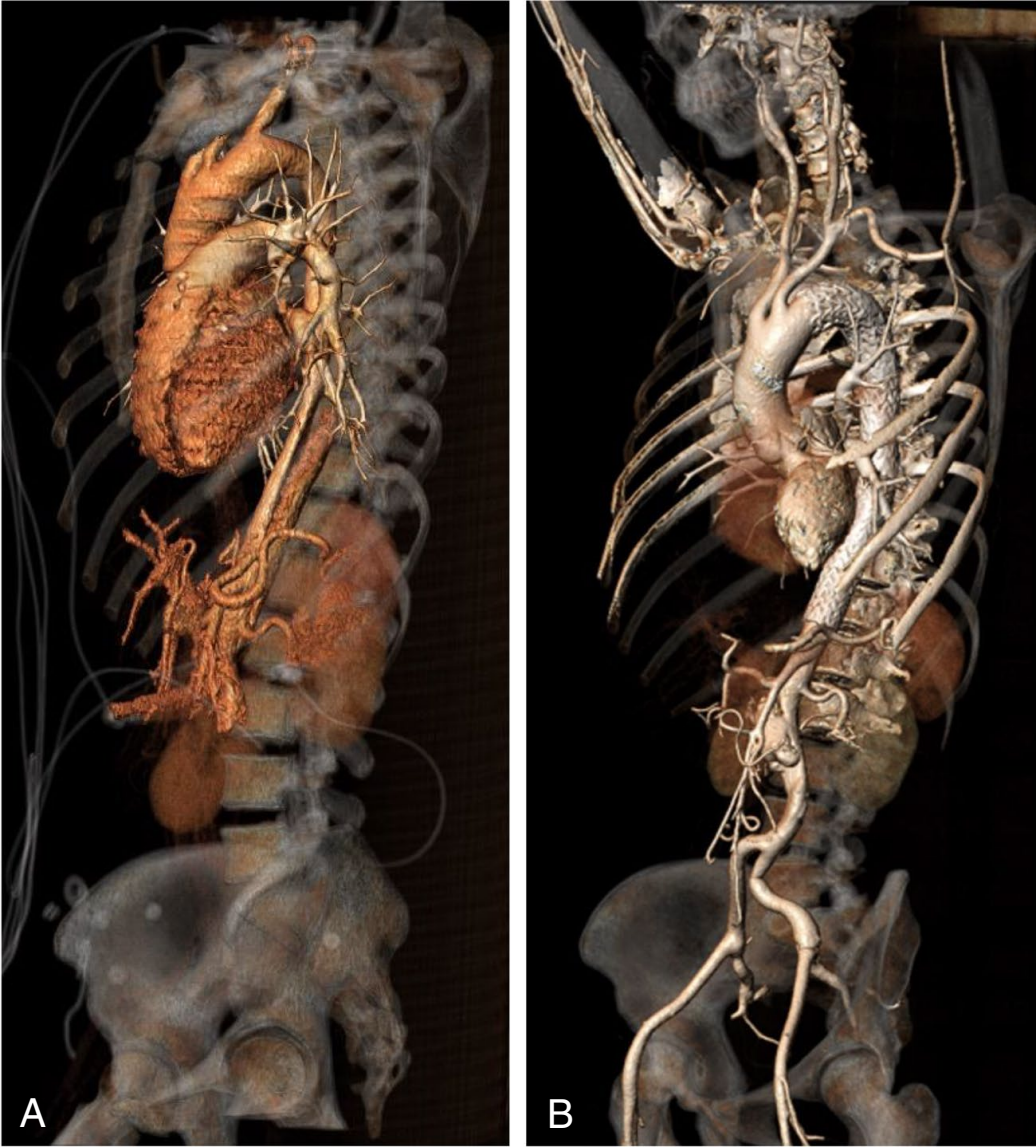
Preoperative (**A**) and 13 years after TEVAR (**B**) 3D reconstruction of the CTA of the thoracic aorta. 3D, three-dimensional; CTA, computed tomography angiography; TEVAR, thoracic endovascular aortic repair

After 5 years, FU scans were mainly performed by magnetic resonance angiography (MRA) as shown in Fig. 2. Until today, 13 years after the onset of disease, the FU was uneventful with no signs of diameter progression, endoleak, migration, or material fatigue and an excellent morphological result with only a small persistent perfusion of the infrarenal aortic segment and complete aortic remodeling. The three-dimensional reconstruction (3D reconstruction) of the CTA performed 13 years after TEVAR is shown in part B of Fig. 3.

Overall, there was no need for major reinterventions.

## Discussion

This case shows that treating type B aortic dissection by covering only the thoracic aortic segment can induce complete aortic remodeling despite persisting downstream dissection. In individual cases with a suitable morphology (e.g., one isolated main entry), this may lead to aortic stabilization without late FL expansion, need of aortic reinterventions, or adjunct procedures.

Several studies showed that TEVAR alone can lead to a stable disease without late expansion of the abdominal aortic segment. Nassib et al. reported in 2021, in a cohort of 41 patients, that late expansion of the abdominal aorta can be prevented by TEVAR alone in about 96% [2].

However, a debate exists regarding the need of adjunct coverage of the abdominal aortic segment, e.g., with bare metal stents like the PETTICOAT technique.

This procedure describes an extension of the thoracic covered stent graft with bare metal stents distally into the visceral segment to prevent secondary FL expansion due to persistent FL perfusion.

A recent meta-analysis by Bertoglio et al. shows a significant advantage in combinated proximal TEVAR + distal bare metal stenting (PETTICOAT technique) versus TEVAR alone in thoracic true lumen expansion. However, there was no difference regarding the aortic remodeling [3].

No matter which approach is chosen, device durability is of utmost importance. In 2009, the then new CTAG form Gore ® received CE mark approval for TEVAR in Europe. The described patient was the first in our center treated with the new generation CTAG. The new generation had improved conformability and adaptable radial force with respect to the underlying aortic pathology. In 2015, an in-house study showed a 100% apposition to inner wall independent from aortic arch type [4].

The 10-year experience with the Gore® CTAG published in 2022 showed a very good durability and a favorable long-term outcome. The rate of reinterventions was low during these 10 years (7.2% overall reinterventions), as was the migration rate of only 2.6%. No fatigue, no infection, nor obstruction were observed [5]. Skrypnik et al. stated that the device can be regarded as persistently safe and durable up to long-term FU while treating plenty of different thoracic aortic pathologies. Nevertheless, it must be considered that only 7 of the 194 patients were actually followed up for about or even more than 8 years. This case shows very well the described course in the summarized data based on a long followed up individual course.

Long-term FU adherence is rare in patients with thoracic aortic disease. This is mostly due to relevant mortality rates of aortic dissections as well as severe comorbidities in degenerative aortic disease. Yet, aortic dissections represent a chronic and serious disease requiring close FU at an expert center, due to the high risk of secondary and late complications during long-term course.

Our institutional FU-protocol tries to keep patients adherent by performing a CTA postoperative before discharge, clinical examination, and CTA after the first year and every following year thereafter. Especially in younger patients, this is of high importance to be able to react in time, but on the other hand, it also allows to see long-term FU in individual cases with very positive results. To prevent high radiation exposures in the long term, after stable follow-up over the first 3–5 years, resort should be made to MRA, which is more artifact-prone and more costly, but radiation-free.

## Conclusion

With contemporary devices and adapted therapy, TEVAR seems able to treat complex thoracic disease. The CTAG appears to be a prosthesis with long durability, although reliable long-term data are lacking currently.

The pathology presented here is a suitable indication for TEVAR when treated by experienced surgeons and considering individual anatomical characteristics. Long-term FU is mandatory to verify efficacy and efficiency of both treatment and monitoring of aortic diseases to identify adverse events in a timely manner.

## Data Availability

The Data is not available to be shared.
